# Improving cancer symptom awareness and help-seeking among adults living in socioeconomically deprived communities in the UK using a facilitated health check: A protocol for the Awareness and Beliefs About Cancer (ABACus) Randomised Control Trial

**DOI:** 10.1186/s12889-019-6612-9

**Published:** 2019-03-11

**Authors:** Yvonne Moriarty, Julia Townson, Harriet Quinn-Scoggins, Louise Padgett, Sioned Owen, Stephanie Smits, Rebecca Playle, Polyxeni Dimitropoulou, Bernadette Sewell, Vasiliki Kolovou, Peter Buckle, Ben Carter, Adrian Edwards, Julie Hepburn, Maura Matthews, Caroline Mitchell, Richard D Neal, Michael Robling, Fiona Wood, Kate Brain

**Affiliations:** 1 Centre for Trials Research, Cardiff University, Neuadd Meirionnydd, Heath Park, Cardiff, CF14 4YS UK; 2 Division of Population Medicine, School of Medicine, Cardiff University, Neuadd Meirionnydd, Heath Park, Cardiff, CF14 4YS UK; 3 Leeds Institute of Clinical Trials Research, University of Leeds, Leeds, LS2 9JT UK; 4 Tenovus Cancer Care, Gleider House, Ty-Glas Rd, Cardiff, CF14 5BD UK; 5 Swansea Centre for Health Economics, College of Human and Health Sciences, Swansea University, Singleton Park, Swansea, SA2 8PP UK; 6 Public Involvement Community, Health and Care Research Wales support Centre, Castlebridge 4, 15-19, Cowbridge Road East, Cardiff, CF11 9AB UK; 7 Institute of Psychiatry, Psychology and Neuroscience, King’s College London, De Crespigny Park, London, SE5 8AF UK; 8 Academic Unit of Primary Medical Care, University of Sheffield, Northern General Hospital, Sheffield, S5 7AU UK; 9 Leeds Institute of Health Sciences, University of Leeds, Worsley Building, Clarendon Way, Leeds, LS2 9NL UK; 10 Marie Curie Research Voices, Marie Curie, 89 Albert Embankment, London, SE1 7TP UK

**Keywords:** Cancer Awareness, Behaviour Change, Help-seeking, Deprived Communities, Randomised Control Trial

## Abstract

**Background:**

Cancer survival is lower in socioeconomically deprived communities, partly due to low awareness of symptoms, negative beliefs and delayed help-seeking. We developed an interactive health check questionnaire facilitated by trained lay advisors. It entails 29 questions about background, lifestyle and health with tailored behaviour change advice. Personalised results are printed using a traffic light (red/amber/green) system, highlighting areas where action should be taken. This is an individually randomised control trial to test effectiveness of the health check on symptom recognition.

**Methods:**

A total 246 participants aged 40+ years will be recruited from community and healthcare settings in socioeconomically deprived areas of Yorkshire and South Wales. Participants will be randomised to receive the health check or standard care (1:1 ratio). Outcome measures include: adapted Awareness and Beliefs about Cancer (primary outcome), brief State Trait Anxiety Inventory, intentions and motivation to adopt recommended health behaviours (early symptom presentation, cancer screening and lifestyle behaviours), adapted Client Service Receipt Inventory, brief medical history/screening and demographic questionnaire at: baseline; 2-weeks; and 6-months post-randomisation. A purposive sample of intervention sessions will be audio-recorded (*n* = 24) and half will additionally be observed (*n* = 12). Semi-structured interviews will take place at 2-weeks (*n* = 30) and 6-months (*n* = 15–20) post-randomisation. The primary analysis will compare cancer symptom recognition scores between arms at 2-weeks. Secondary analysis will assess cancer beliefs, barriers/time to presentation, screening and lifestyle behaviours, anxiety and costs. A process evaluation will assess intervention fidelity, dose and contamination.

The London-Surrey NHS Research Ethics Committee (Ref: 17/LO/1507) approved this trial.

**Discussion:**

This is a trial of a theoretically underpinned complex intervention which has undergone phase 1 and 2 development work. The findings will evaluate evidence about the effect of the health check on symptom awareness. Although there are few exclusion criteria there are limitations regarding the population we are able to reach, who may have even higher risks of late diagnosis and poor cancer prognosis. However, the health check has the potential to improve cancer symptom awareness and encourage early help-seeking behaviour in deprived populations, thereby reducing inequalities in longer term cancer outcomes.

**Trial Registration:**

Retrospectively registered with ISRCTN (Ref:ISRCTN16872545) on 12.01.2018.

## Background

Cancer outcomes are considerably poorer in the most socioeconomically deprived areas of the UK [1–3], including West and South Yorkshire and South Wales. This reflects high risk lifestyle behaviours (e.g. smoking, poor diet, low exercise) which have been linked to increased risk of developing cancer [4]. Evidence suggests that low awareness of cancer symptoms, fear of cancer or fatalistic beliefs about cancer and concerns over wasting the doctor’s time are higher in lower socioeconomic groups [5] resulting in delayed help seeking [5–8], in turn leading to late stage diagnosis and lower uptake of cancer screening [9]. There is a need for interventions which overcome this, by addressing negative beliefs about cancer and highlighting the benefits of early diagnosis, as well as raising awareness of potential cancer symptoms, in order to reduce the time to symptom presentation and improve long term treatment outcomes.

Mass media awareness campaigns have been widely used across public health with the aim of changing a range of health behaviours (including increasing cancer knowledge and symptom recognition and encouraging help seeking behaviour) [10]. Emerging evidence suggests that targeted, intensive community-based behaviour change interventions may be more successful at improving cancer awareness in high risk disadvantaged populations [11–15]. In particular, interventions that draw on pre-existing social networks and social influences [16] have the potential for more successful outcomes in this context. Involving trusted and trained lay advisors may be a successful method for engaging and delivering cancer messages in a compassionate and non-judgmental manner to people living in deprived communities [11, 17]. However, the Improving Rural Cancer Outcomes (IRCO) trial of a community-based symptom awareness and GP educational intervention in rural Western Australia did not observe a significant effect of the intervention on time to symptom presentation [18]. The authors suggest that this may reflect limited intervention “dose”. It is therefore important to consider the optimal dose and intensity of cancer awareness interventions targeted at high risk disadvantaged populations.

While previous targeted community-based complex interventions that use evidence-based behaviour change techniques (BCTs) are promising in encouraging cancer awareness and earlier help-seeking [12–14], high quality evidence is needed to test intervention effectiveness in ‘real life’ settings. The ABACus 3 trial is testing a tailored health check intervention facilitated by a lay advisor in deprived communities in South and West Yorkshire and South Wales.

### Health Check intervention

**Fig. 1:**
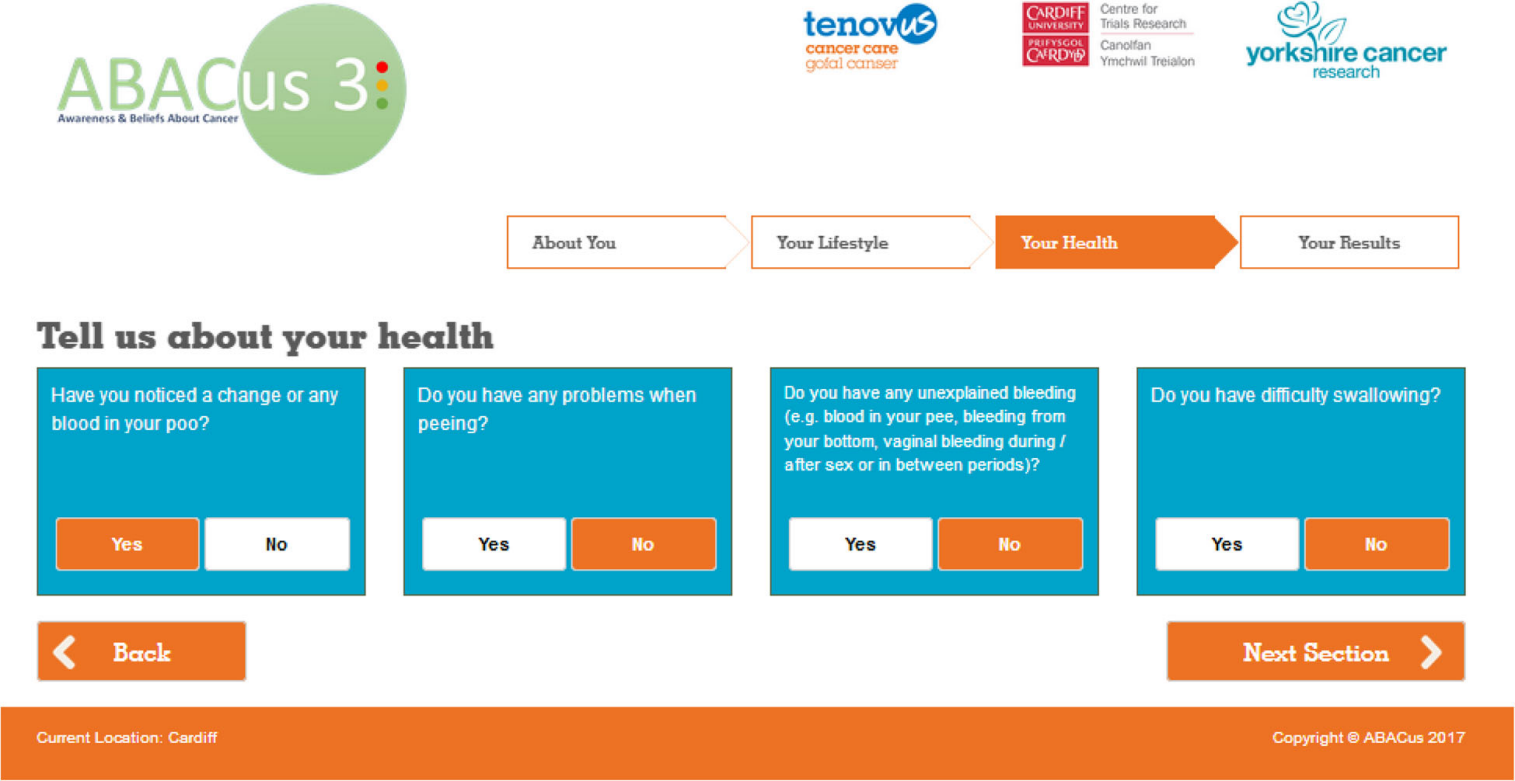
Screen shot of the ABACus Health check ‘Your Health’ Section

We developed and piloted a community outreach health check intervention facilitated through a lay advisor, designed to improve cancer symptom knowledge, encourage positive beliefs in relation to early cancer detection, and increase motivation to seek help among adults living in deprived communities [16, 19]. The health check has been informed by a theoretical understanding of the barriers and enablers to timely help-seeking among people living in disadvantaged communities [19–21], and comprises an interactive touchscreen questionnaire with behavioural support delivered face-to-face by a trained lay advisor (see Fig. 1 for screenshot of the touch screen questionnaire). The intervention is primarily designed to reduce the “patient interval”, defined as the time between appraising a bodily change as a potential symptom of cancer and presenting in primary care [7]. It also attempts to integrate early symptom detection with cancer screening and cancer prevention recommendations, by including content relating to cancer symptoms, screening, and risk factors (i.e. smoking, diet and inactivity).

In line with the Medical Research Council guidance on developing and evaluating complex interventions [22], two phases of development and feasibility/pilot testing were undertaken in partnership with local stakeholder groups. Findings from phase 1 suggested that the health check is beneficial and acceptable to members of the public, health professionals and community partners living and working in deprived areas of South Wales [19]. Results from phase 2 demonstrated the feasibility and acceptability of recruiting people from healthcare and community settings within disadvantaged communities to undertake the health check [16].

The health check comprises 29 questions divided into three main sections; *‘About You’*, *‘Your Lifestyle’* and *‘Your Health’*. Questions are tailored according to participants’ age and gender (See Table 1 for a breakdown of the questions). Individualised results are provided in a ‘*Results*’ section and are displayed using a traffic light system, with ‘green’ indicating results where no signposting or change is suggested, ‘amber’ indicating an area where signposting or change could be considered, and ‘red’ results indicating that action should be taken. Information and signposting to relevant services (for example, stop smoking and weight loss services) are provided by the lay advisors, based on individual results and tailored to local availability. The intervention manual details all written and verbal information to be provided to participants for each response type across each question. It also maps out each of the BCTs to be used within each section. Lay advisors are extensively trained to deliver the intervention and are formally assessed to ensure familiarity with all components of the intention (i.e. ‘About You’, ‘Lifestyle’, ‘Your Health’) and associated BCTs tailored according to individual responses.

**Table 1:** Interactive Health Check Questions

Sections	Questions
Section 1: About You	Have you ever been diagnosed with cancer?
	Do you have 2 or more close relatives who have been under the age of 50 when diagnosed with cancer?
	Please give your height in Feet and Inches / Centimetres Please give your weight in Stones and Pounds/Kilos
	Did you receive your bowel screening kit in the post? If yes, did you send it back?
	Have you been invited for a cervical smear test? If yes, did you attend?
	Have you been invited for your breast screening test? If yes, did you attend?
Section 2: Your Lifestyle	Do you smoke? If yes, On average How many cigarettes a day do you smoke
	Are you exposed to another person’s smoke on a regular basis?
	Do you drink alcohol? If yes, on average How many units of Alcohol do you drink each week?
	On average, how many hours a week do you exercise in total, adding up any daily amounts?
	How often do you eat 5 portions of fruit and vegetables in a day?
Section 3: Your Health	Do you have a cough that won’t go away? If yes, do you bring up blood when you cough?
	Have you noticed any unusual lumps on your body (e.g. breasts, testicles, armpits, groin)?
	Have you noticed a change in how your skin looks (e.g. change to a mole, freckle or patch of skin)?
	Do you have a sore or ulcer in your mouth that will not heal?
	Have you noticed a change or any blood in your poo?
	Do you have any problems when peeing?
	Do you have any unexplained bleeding (e.g. blood in your pee, bleeding from your bottom, vaginal bleeding during /after sex or in between periods)?
	Do you have difficulty swallowing?
	Have you been losing weight without trying to?
	Have you noticed any unexplained change in your appetite?
	Do you feel tired most of the time?
	Do you have an unexplained pain that won’t go away?

### Aim and Objectives

The aim of the trial is to evaluate the effectiveness of a community-based cancer awareness intervention in socioeconomically deprived communities. Specific objectives are to:

1. Test the effects of the health check on cancer symptom awareness and help-seeking behaviour among adults living in socioeconomically deprived communities in South and West Yorkshire and South-East Wales.
2. Evaluate the costs associated with the health check and estimate the cost-effectiveness of the intervention.
3. Assess whether the intervention was delivered as intended, whether there are any contaminating factors to understand the mechanisms of change.

## Methods

### Patient and Public Involvement

The trial team includes two patient/public research partners (one of whom was also involved in the phase 2 study) who provide support and knowledge at every stage of the research process. One partner lives in one of the target recruitment areas, the other has significant family associations with the other area. Both have been affected by cancer.

The research partners have been critical for the early development and set-up of the trial and have provided detailed input to the protocol development. They additionally contribute to all public facing materials (i.e. information booklet content and design, questionnaire design etc.), generate ideas on how best to engage the target population, support data interpretation as well as provide ideas on dissemination opportunities.

### Trial design and setting

**Fig. 2:**
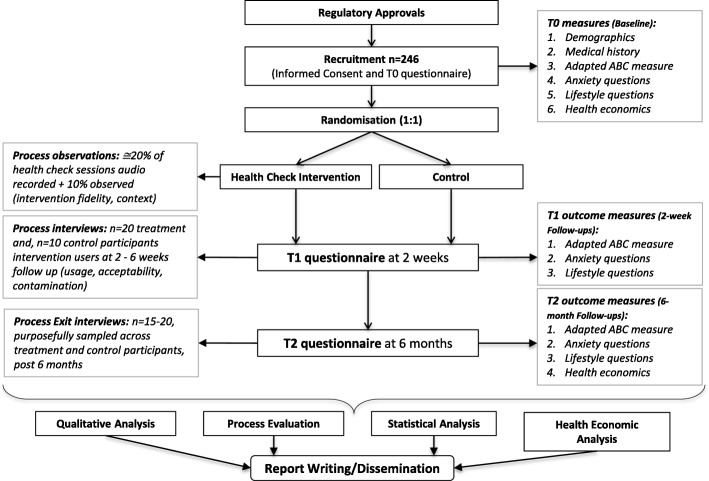
Trial Flow diagram

This is an unblinded individually Randomised Controlled Trial of an online Cancer Health Check intervention with additional tailored verbal information delivered by a lay advisor in areas of high deprivation in South and West Yorkshire (i.e. Sheffield, Wakefield, Barnsley, Doncaster, Rotherham etc.) and South-East Wales (i.e. Merthyr Tydfil, Newport etc.) UK. See Fig. 2 for a flow diagram of the trial design.

Areas of high deprivation are identified using the Index of Multiple Deprivation (IMD) for England [23] and the Welsh Index of Multiple Deprivation (WIMD) for Wales [24]. Settings include community (local community groups, one-to-one community sessions, community events) and healthcare (GP practices and community pharmacies) venues.

This protocol has been drafted in accordance with the SPIRIT guidelines [25].

### Participant selection

All participants must meet the following eligibility criteria.

Inclusion criteria:

- Aged 40 years and over.
- Recruited from socioeconomically deprived areas (i.e. lowest quintile) of South and West Yorkshire (i.e. Sheffield, Barnsley, Rotherham, Wakefield) as measured by the IMD [23] or South-East Wales (i.e. Merthyr Tydfil and Newport) as measured by the WIMD [24].

Exclusion criteria:

- Non-English speakers.
- Unable to give written informed consent (as defined by Good Clinical Practice [26]).
- A participant from the phase 2 study.

### Outcomes

The primary outcome is cancer symptom knowledge [27] including specific (e.g. rectal bleeding, unusual lump) and non-specific (e.g. tiredness, unexplained weight loss) symptoms measured at two weeks post-randomisation.

Secondary outcomes include: cancer beliefs [27]; barriers to presentation [27, 28]; help-seeking intentions [27]; state anxiety [29]; intervention implementation costs; cost-effectiveness; intentions and confidence to adopt recommended health behaviours where relevant (smoking, physical activity, weight loss, fruit and vegetable intake, alcohol consumption, screening attendance, symptom presentation) [4, 30].

### Measures

The following measures will be used:

- Adapted Awareness and Beliefs About Cancer questionnaire (ABC) [27].
  (The ABC measure was adapted to be used with this population during phase 2 and has been further refined following phase 2 findings. Changes made mainly focus on phrasing and wording of questions to ensure they are clearly understood by the target population.)
- Six-item short-form State-Trait Anxiety Inventory (STAI-6) [29].
- Client Service Receipt Inventory.
- Behavioural intentions and confidence questionnaire developed using Theory of Planned Behaviour [30].

### Participant Identification & Recruitment

Participants will be identified and recruited by the lay advisors with support from local site staff and/or local research nurses/officers where possible. Recruitment days will be arranged in advance and will take place at local facilities within communities identified as areas of high socioeconomic deprivation (using the IMD and WIMD).

A total of 246 participants will be recruited to the trial, with two thirds (*n* = 164) of participants recruited from South/West Yorkshire and one third (*n* = 82) recruited from South-East Wales over a 15 month period. This reflects strategic priorities of the funder to address the needs of the Yorkshire population, while recognising similarities in the sociodemographic characteristics of the populations across the two geographical sites.

Venues will provide access to a private room to ensure participant privacy and confidentiality. Two recruitment methods will be used: Route 1 - pre-booked appointments, and Route 2 – opportunistic. Wherever possible, pre-booked appointments (with support from local staff) will be used to book in interested individuals. Opportunistic recruitment will be used where local staff are unable to support the appointment system. In such instances, lay advisors will either visit venues a few days in advance to approach individuals and book them in for the upcoming recruitment day or approach potential recruits on the day or a combination of both.

### Screening, registration and consent

The lay advisors will initially check participant eligibility verbally when individuals show an interest in the study and if confirmed as eligible will then proceed to full recruitment. The study will be explained to individuals with the support of the participant information booklets. Those individuals who proceed to recruitment will be asked to provide written informed consent at which point their eligibility will be formally confirmed (using a checklist) by the lay advisor. We will additionally seek consent from participants at recruitment to take part in the qualitative aspects of the trial (if relevant) and to be contacted in the future should a related follow-on study take place.

### Randomisation

Participants will be individually randomised to either intervention (facilitated health check) or control (usual available care/support) in a 1:1 ratio. Randomisation will occur immediately following baseline data collection and participants will be informed by the lay advisor of their allocation. A computerised random number sequence will be generated to indicate group allocation. This will be facilitated through a bespoke database. Lay advisors will be blind to the randomisation sequence in order to minimise any selection bias during recruitment and data collection. Only the direct trial team will have access to this information for back-up randomisation purposes.

### Data collection

Table 2 provides a breakdown of data collection across time points. A data management plan will outline how all data will be collected, managed and stored. All data will be managed in strict confidence according to GDPR (EU 2016/679).

**Table 2:** Enrolment/Assessments schedule [42]

Procedures/Time point	Set-up	Screening	Baseline	Intervention	Follow-up	
	- 1 month	B (over 12 months)			+ 2 weeks	+ 6 months
Recruitment						
Eligibility assessment		X				
Informed consent			X			
Contact details form			X			
Randomisation			X			
Data collection						
Demographic questionnaire			X			
Medical history questionnaire			X			
Adapted ABC measure			X		X	X
Cancer worry			X		X	X
Lifestyle questionnaire			X		X	X
Resource use			X			X
Intervention Delivery						
Lay advisor training	X					
Intervention delivery or control				X		
Process measures						
Interviews with HCAs	X				X	
Site summary logs		X				
Lay advisor timesheets		X	X	X		
Observation/Audio recording of Intervention delivery				X		
Participant Interviews (2–6 weeks)					X	
Participant exit interviews (post trial)						X

Participants will complete the adapted ABC questionnaire at baseline, 2 weeks and 6 months post-randomisation. Baseline data will be collected electronically via an IPad and entered directly onto the bespoke trial database. Follow-up data will primarily be collection on the phone and inputted directly on the database, however if participants cannot be contacted after four attempts, paper case report forms will be sent via the post.

To support the process evaluation, a purposive sample (based on setting type, age and gender) of participants will be interviewed at 2–6 weeks post intervention delivery (*n* = 30) and post-trial (*n* = 15–20). A purposive sample (based on setting type, age and gender) of health check sessions (20%, *n* = 24/123) will be audio-recorded and half of these (10%, *n* = 12/123) will be purposive sampled (based on setting type, age and gender) to be additionally observed. Lay advisors will be interviewed before beginning participant recruitment and at the end of recruitment (n = 3 + 3). Site summary logs will be completed for each recruitment day to record further process data.

### Incentives

As a thank you for their time, participants will be offered high street shopping vouchers to the value of £15. They will receive a £10 voucher after completing the baseline questionnaire and a £5 voucher after completing the 6-month questionnaire. A sample of participants will be offered a further £10 voucher for taking part in an interview.

### Process evaluation

In parallel to the trial, a process evaluation will be conducted following the MRC guidance [31] to assess: fidelity (whether the intervention has been delivered as intended and as a measure of quality assurance); dose (whether tailored BCTs were delivered according to participant self-reported behaviours during the health check); contamination (whether there were any external factors that may have influenced participant behaviour/responses); reach (whether the target population received the intervention); key mechanisms of change (which components of the intervention may have caused change i.e. mapped BCTs according to domain questions e.g. increased symptom recognition as a results of increased capacity (i.e. specific knowledge and BCTs: *information about health consequences*)). The evaluation will draw on a combination of qualitative (observations and interviews) and quantitative (recruitment numbers, resource use, manual adherence) data.

Fidelity of adherence to the intended intervention delivery and tailored BCTs dose delivery will be assessed in the process evaluation. A sample of sessions (20%, *n* = 24/123) will be audio-recorded and half of these (*n* = 12/123) will additionally be observed to ascertain the extent to which the intervention was delivered according to the manual and to assess the levels of exposure. A structured coding framework will be developed to support the assessment of fidelity and dose.

### Sample size

A sample of 246 participants is required in order to achieve 90% power to detect an effect size of 0.5 in the primary outcome of cancer symptom recognition, using a two-sided test and 5% significance threshold, and assuming 30% attrition at two weeks follow-up. An effect size of this magnitude equates to intervention participants recognising on average one extra cancer symptom during the follow-up period (SD = 2.2) [32].

### Quantitative Analysis

#### Adapted ABC questionnaire validation

The baseline data from the adapted ABC questionnaire will be used to explore properties such as item functioning, responsiveness, validity and reliability. Factor analysis and summary statistics will be used to determine internal consistency and item correspondence to constructs underlying the outcome measures.

#### Descriptive

Descriptive statistics of demographic variables (age, gender, ethnicity, marital group, access to health care, presence of comorbid conditions, socioeconomic indicators, i.e. educational attainment and occupational status) and outcomes for baseline and follow-up, split by treatment arm, will be presented to summarise the unadjusted data. The response rate will be similarly summarised. Assessment of drop-out bias will not be tested but will tabulate the baseline demographic descriptives of those completing and those not completing the 2 week follow-up.

#### Main analyses

The primary analysis will be on a complete case basis following intention-to-treat principles, and will determine the difference in ABC score between the two arms at 2 weeks, including baseline cancer symptom recognition as a covariate. The distributional assumptions of the linear model will be checked by visual inspection of fitted versus residuals plots. The primary outcome data will be transformed where appropriate. If they remain non-normal, bootstrapping will be used to generate regression coefficients and confidence intervals. The mean (SD) ABC for the control and intervention groups at baseline and follow-up will be tabulated. The primary outcome effect will be reported unadjusted and adjusted for baseline ABC, with 95% CI and *p*-value. Covariates to be considered for inclusion in the model are age, gender, recruitment setting and socio-economic group (as measured by area level deprivation using the IMD [23] and WIMD [24]. Secondary outcomes will be analysed similarly. Proposed sensitivity analyses include the investigation of missing data and the use of multiple imputation for the primary outcome. We will also investigate the effect of collection time of the primary outcome (within/without the specified time window). Pre-specified interactions of interest are age and gender with the intervention. A detailed Statistical Analysis Plan (SAP) will be completed and signed off before the database is locked and analysis begins. The trial statistician will be blind to participant allocation during analysis. Study results will be reported in line with the CONSORT statement [33].

The intervention will be delivered by three health check advisors. Any clustering by advisor in the intervention arm will be investigated via a partial cluster model of the primary outcome [34, 35]. The adjusted effect for clustering (by advisor) with 95% CI, p-value and Intra-Cluster Correlation will be reported. If the distributional assumptions for linearity are not met this analysis will use the binary primary outcome and generalized modelling.

### Qualitative Analysis

Qualitative data (observations, session recordings, participant interviews and lay advisor interviews) will be transcribed and anonymised for analysis. Thematic and content analysis will be used. Where possible, data will be triangulated and evidence derived from the process evaluation will be used to inform which aspects of the health check might make a change in relation to the outcomes. Details of the analysis to be conducted are outlined below. A Qualitative data collection and Analysis Plan (QAP) will be developed and signed off in advance of data collection and will be regularly reviewed during data collection and analysis.

#### Observations of health check sessions

Observations/audio recordings will be analysed for two main purposes. Firstly, we will ascertain fidelity by content coding anonymised transcripts of health check sessions for evidence of the BCTs identified in the health check manual [19, 36]. A fidelity definition and acceptable range will be agreed upon by the team in advance. Two researchers will be involved in coding transcripts independently and a subset will be double-coded to assess inter-rater reliability. Fidelity will be assessed using content analysis by:

- comparing manual-specified BCTs with the number of BCTs delivered in health check sessions;
- comparing delivery of BCTs according to session duration, advisor and site;
- examining discrepancies between health check advisors’ self-reported coverage and actual coverage of BCTs, and delivery of any BCTs that are not stipulated in the health check manual.

Secondly, we will carry out further inductive thematic analysis [37] of the audio-recorded health check sessions to gain a deeper understanding of social context and constructions of cancer awareness and help-seeking. Dual coding will be undertaken to reduce potential bias [37].

#### Interviews

Anonymised participant interview transcripts and lay advisor paired interviews will be analysed thematically [37], with 20% double coded (to ensure consistent understanding of code definitions and application between coders), to explore which aspects of the health check were perceived to be most useful, why and participants’ potential experiences of wider contextual contamination (i.e. other cancer awareness campaigns).

### Health Economic Analysis

The trial will include a health economic evaluation from an NHS perspective to provide an estimation of costs and cost-effectiveness of the health check intervention. No discounting will be applied as the length of intervention and follow-up do not exceed one year.

Implementation cost of the intervention will be calculated from resource use and standard unit costs (where available) as well as financial records. Resource use associated with the intervention will be established from site summary forms and through interviews with the lay advisors. The main components are expected to be lay advisor training, supervision and support, and advisor time and travel. As the intervention will be provided in routine health care and non-medical community support settings, we do not anticipate additional costs to the participants and will therefore not collect out-of-pocket expenses as part of the implementation cost. Healthcare resource use will be measured using an adapted Client Service Receipt Inventory to compare differences at baseline and six months.

The implementation cost will be compared to the outcomes of the trial in tabular form as part of a cost-consequences analysis that will allow comparison of costs to the main trial outcomes. We do not expect any short-term effect of the intervention on participants’ quality-of-life and will not collect these data as part of the health economic analysis. Cost-effectiveness analyses will be conducted for clinical outcomes where statistically significant differences were found. These analyses will calculate the cost per point improvement (e.g. in cancer symptom awareness, anxiety etc.) and present the results as incremental cost-effectiveness ratios. Uncertainty will be assessed using one-way deterministic and probabilistic sensitivity analyses with key parameters varied within plausible ranges (e.g. 95% CIs) and cost-effectiveness acceptability curves will be presented.

If feasible, we will develop a simple decision-analytic model to extrapolate costs and outcomes to a longer-term horizon using the intervention costs and trial outcomes. These will be supported by data available in the published literature reporting health outcomes in relation to cancer symptom awareness. Feasibility of the modelling exercise will depend on data availability in the public domain and the trial outcomes. If the trial were not to find any significant differences in the primary or secondary outcomes, modelling will be considered non-feasible. A review of the relevant literature will be conducted to inform the model. Deterministic and (if feasible) probabilistic sensitivity analyses will be conducted to account for the uncertainty in key parameters informing the analysis.

### Ethics

This trial has been assessed as low risk. No adverse events are being collected due to the short contact period with each participant and the potential for this to create unnecessary burden on participants. Informed consent is taken from each participant at recruitment. Where a participant has not consented to take part in a qualitative interview, intervention delivery audio recording or observation they will not be approached regarding these aspects of the trial.

Some participants may find it difficult to talk about cancer, may find questions about cancer symptoms uncomfortable to discuss or may feel embarrassed about their lifestyle. When dealing with participants, researchers will be mindful that questions could be upsetting and that the social determinants of health, including the person’s environment, are important drivers of behaviour [38] alongside self and societal stigma relating to obesity, smoking and alcohol use [39, 40].

Research staff are experienced in collecting sensitive data and specific training is provided to support staff. In order to provide participants with privacy to discuss their responses with the lay advisor, the baseline assessments take place in a private room. Participants are reminded that their participation is highly confidential and any information they share will not be shared with other parties. Follow-up phone assessments are scheduled at a time suitable to participants which allows them to find a private space to talk. However, if at any point any participant becomes upset they are provided with support and, where appropriate, additional support services are highlighted to them. Participants are additionally offered a break and/or are able to continue their participation at a different date/time if they wish.

Research data will be held for 15 years and archived securely. This is in line with Cardiff University policies.

### Dissemination

Study results will be disseminated widely through academic, clinical, policy and community networks and to trial participants. An inclusive publication policy has been developed to support this and provides all members of the team with an opportunity to volunteer ideas and input to planned outputs. The publication policy will be discussed at monthly meetings and any new ideas added.

## Disscussion

This is the first randomised controlled trial of a facilitated behaviour change intervention aimed at improving cancer awareness in socioeconomically deprived communities in the UK. Previous research suggests that tailored community-based interventions delivered by trusted lay advisors could lead to an increase in cancer symptom awareness and encourage help-seeking behaviour among adults living in disadvantaged communities, for whom long-term health may not be a priority due to competing life demands and low socioeconomic resources [39]. High quality evidence is needed to test the effectiveness of such interventions within real life settings. The current trial follows on from theory modelling [19] and feasibility testing [16], according to the MRC guidance on developing and evaluating complex interventions [22]. We aim to reach individuals in deprived areas who experience the poorest long-term cancer outcomes. However, despite broad inclusion criteria, the trial excludes individuals who lack capacity to provide informed consent and those who do not speak English, who may have the highest need. In addition, it is not designed to reach members of the community who do not or who are unable to (e.g. people who are housebound) attend the community venues that are targeted within this trial. Arguably, these populations may have higher needs and may be even harder to reach and engage [41]. Future research could consider how to extend reach to these populations within disadvantaged communities.

## Conclusion

The findings of the trial will be critical to informing effective methods of engaging high risk disadvantaged populations in cancer awareness, with the potential to reduce socioeconomic inequalities in cancer outcomes and the possibility of wider implementation across the UK.

## Abbreviations

ABACus: Awareness and Beliefs About Cancer trial
ABC: Awareness and Beliefs about Cancer measure
BCT: Behaviour Change Techniques
CI: Confidence Intervals
CONSORT: CONsolidated Standards Of Reporting Trials
GDPR: General Data Protection Regulation
GP: General Practitioner
HCA: Health Check Advisor (lay advisor)
IMD: Index of Multiple Deprivation
ISRCTN: International Standard Randomised Controlled Trial Number
MRC: Medical Research Council
NHS: National Health Service
QAP: Qualitative data collection and Analysis Plan
SAP: Statistical Analysis Plan
SD: Standard Deviations
SPIRIT: Standard Protocol Items: Recommendations for Interventional Trials
STAI-6: Six-item short-form State-Trait Anxiety Inventory
WIMD: Welsh Index of Multiple Deprivation

## References

[1] Ellis L, Coleman MP, Rachet B (2012). How many deaths would be avoidable if socioeconomic inequalities in cancer survival in England were eliminated? A national population-based study, 1996–2006. Eur J Cancer.

[2] Lyratzopoulos G, Abel GA, Brown CH, Rous BA, Vernon SA, Roland M, et al. Socio-demographic inequalities in stage of cancer diagnosis: Evidence from patients with female breast,lung, colon, rectal, prostate, renal, bladder, melanoma, ovarian and endometrial cancer. Ann Oncol 2013;24(3):846–850. 10.1093/annonc/mds526 PMC3574550 23149571

[3] McPhail S, Johnson S, Greenberg D, Peake M, Rous B (2015). Stage at diagnosis and early mortality from cancer in England. Br J Cancer.

[4] Brown KF, Rumgay H, Dunlop C, Ryan M, Quartly F, Cox A (2018). The fraction of cancer attributable to modifiable risk factors in England, Wales, Scotland, Northern Ireland, and the United Kingdom in 2015. Br J Cancer.

[5] Quaife SL, Winstanley K, Robb KA, Simon AE, Ramirez AJ, Forbes LJL (2015). Socioeconomic inequalities in attitudes towards cancer: An international cancer benchmarking partnership study. Eur J Cancer Prev.

[6] Macleod U, Mitchell ED, Burgess C, Macdonald S, Ramirez AJ (2009). Risk factors for delayed presentation and referral of symptomatic cancer: Evidence for common cancers. Br J Cancer.

[7] Walter F, Webster A, Scott S, Emery J (2012). The Andersen Model of Total Patient Delay: A systematic review of its application in cancer diagnosis. J Heal Serv Res Policy.

[8] McCutchan GM, Wood F, Edwards A, Richards R, Brain KE. Influences of cancer symptom knowledge, beliefs and barriers on cancer symptom presentation in relation to socioeconomic deprivation: A systematic review. BMC Cancer. 2015;15(1000). 10.1186/s12885-015-1972-8 PMC4688960 26698112

[9] Hiom SC (2015). Diagnosing cancer earlier: reviewing the evidence for improving cancer survival. Br J Cancer.

[10] Wakefield MA, Loken B, Hornik RC (2010). Use of mass media campaigns to change health behaviour. Lancet..

[11] McWilliams L, Bellhouse S, Yorke J, Cowan R, Heaven CM, French DP (2018). The acceptability and feasibility of lay-health led interventions for the prevention and early detection of cancer. Psychooncology..

[12] Linsell L, Forbes LJL, Kapari M, Burgess C, Omar L, Tucker L (2009). A randomised controlled trial of an intervention to promote early presentation of breast cancer in older women: effect on breast cancer awareness. Br J Cancer.

[13] Cardarelli K, Jackson R, Martin M, Linnear K, Lopez R, Senteio C (2011). Community-Based Participatory Approach to Reduce Breast Cancer Disparities in South Dallas. Prog Community Heal Partnersh.

[14] Whitaker KL, Scott SE, Wardle J (2015). Applying symptom appraisal models to understand sociodemographic differences in responses to possible cancer symptoms: A research agenda. Br J Cancer.

[15] Austoker J, Bankhead C, Forbes LJL, Atkins L, Martin F, Robb K (2009). Interventions to promote cancer awareness and early presentation: Systematic review. Br J Cancer.

[16] Smith P, Smits S, Owen S, Wood F, Mccutchan G, Carter B (2018). Feasibility and acceptability of a cancer symptom awareness intervention for adults living in socioeconomically deprived communities. BMC Public Health.

[17] McCutchan G, Hiscock J, Hood K, Murchie P, Neal R, Newton G, et al. Engaging high-risk groups in early lung cancer presentation: a qualitative study of symptom presentation and intervention preferences amongst the UK’s most deprived communities. Under Rev. 10.1136/bmjopen-2018-025902 PMC6538016 31122972

[18] Emery JD, Gray V, Walter FM, Cheetham S, Croager EJ, Slevin T (2017). The Improving Rural Cancer Outcomes Trial: A cluster-randomised controlled trial of a complex intervention to reduce time to diagnosis in rural cancer patients in Western Australia. Br J Cancer.

[19] Smits S, McCutchan G, Wood F, Edwards A, Lewis I, Robling M, et al. Development of a Behavior Change Intervention to Encourage Timely Cancer Symptom Presentation Among People Living in Deprived Communities Using the Behavior Change Wheel. Ann Behav Med. 2016:1–15. 10.1007/s12160-016-9849-x PMC6367899 27826697

[20] Cane J, O ‘connor D, Michie S. Validation of the theoretical domains framework for use in behaviour change and implementation research. Implement Sci. 2012;7(37). 10.1186/1748-5908-7-37 PMC3483008 22530986

[21] Michie S, Atkins L, West R (2014). The Behaviour Change Wheel: A Guide To Designing Interventions.

[22] Craig P, Dieppe P, Macintyre S, Michie S, Nazareth I, Petticrew M (2013). Developing and evaluating complex interventions: The new Medical Research Council guidance. Int J Nurs Stud.

[23] English Indices of Deprivation 2015. UK Government [Internet]. [cited 2018 Nov 20]. Available from: https://www.gov.uk/government/statistics/english-indices-of-deprivation-2015

[24] Welsh Index of Multiple Deprivation (WIMP) 2014. Welsh Government [Internet]. [cited 2018 Nov 20]. Available from: http://wimd.wales.gov.uk/

[25] Chan A-W, Tetzlaff JM, Altman DG, Laupacis A, Gøtzsche PC, Krleža-Jeric K (2013). SPIRIT 2013 Statement: Defining Standard Protocol Items for Clinical Trials development of the spirit 2013 STATEMENT. Ann Intern Med.

[26] NIHR Clinical Research Network Coordinating Centre. Good Clinical Practice (GCP) Reference Guide 2016. 3.1. ICH Secretariat, editor. Geneva: ICH Secretariat; 2016. 24 p.

[27] Simon AE, Forbes LJL, Boniface D, Warburton F, Brain KE, Dessaix A, et al. An international measure of awareness and beliefs about cancer: Development and testing of the ABC. BMJ Open. 2012;2(e001758). 10.1136/bmjopen-2012-001758 PMC3547316 23253874

[28] Stubbings S, Robb K, Waller J, Ramirez A, Austoker J, Macleod U (2009). Development of a measurement tool to assess public awareness of cancer. Br J Cancer.

[29] Marteau TM, Bekker H (1992). The development of a six-item short-form of the state scale of the Spielberger State—Trait Anxiety Inventory (STAI). Br J Clin Psychol.

[30] Francis JJ, Eccles MP, Johnston M, Walker A, Grimshaw J, Foy R (2004). Constructing questionnaires based on the theory of planned behaviour: A manual for health service researchers.

[31] Moore GF, Audrey S, Barker M, Bond L, Bonell C, Hardeman W (2015). Process evaluation of complex interventions: Medical Research Council guidance. BMJ..

[32] Forbes LJL, Simon AE, Warburton F, Boniface D, Brain KE, Dessaix A (2013). Differences in cancer awareness and beliefs between Australia, Canada, Denmark, Norway, Sweden and the UK (the International Cancer Benchmarking Partnership): Do they contribute to differences in cancer survival?. Br J Cancer.

[33] Schulz KF, Altman DG, Moher D (2010). CONSORT 2010 Statement: Updated guidelines for reporting parallel group randomised trials. BMJ..

[34] Roberts C, Roberts SA (2005). Design and analysis of clinical trials with clustering effects due to treatment. Clin Trials.

[35] Flight L, Allison A, Dimairo M, Lee E, Mandefield L, Walters SJ (2016). Recommendations for the analysis of individually randomised controlled trials with clustering in one arm - A case of continuous outcomes. BMC Med Res Methodol.

[36] Lorencatto F, West R, Bruguera C, Michie S (2014). A method for assessing fidelity of delivery of telephone behavioural support for smoking cessation. J Cons Clin Psychol.

[37] Braun V, Clark V (2006). Using thematic analysis in psychology. Qualiaitive Res Psychol.

[38] Office for National Statistics. An overview of lifestyles and wider characteristics linked to Healthy Life Expectancy in England: June 2017 [Internet]. 2017. Available from: https://www.ons.gov.uk/peoplepopulationandcommunity/healthandsocialcare/healthinequalities/articles/healthrelatedlifestylesandwidercharacteristicsofpeoplelivinginareaswiththehighestorlowesthealthylife/june2017

[39] Dixon-Woods M, Cavers D, Agarwal S, Annandale E, Arthur A, Harvey J, et al. Conducting a critical interpretive synthesis of the literature on access to healthcare by vulnerable groups. BMC Med Res Methodol. 2006;6(35). 10.1186/1471-2288-6-35 PMC1559637 16872487

[40] Crawford R (1977). You are Dangerous to Your Health: The Ideology and Politics of Victim Blaming. Int J Health Serv.

[41] Bonevski B, Randell M, Paul C, Chapman K, Twyman L, Bryant J, et al. Reaching the hard-to-reach: A systematic review of strategies for improving health and medical research with socially disadvantaged groups. BMC Med Res Methodol. 2014;14(42). 10.1186/1471-2288-14-42 PMC3974746 24669751

[42] Health Research Authority. Protocol guidance and template for use in a Clinical Trial of Investigational Medicinal Product (CTIMP) [Internet]. [cited 2018 Nov 20]. Available from: https://www.hra.nhs.uk/planning-and-improving-research/research-planning/protocol/

